# Galectin-1 Is Part of Human Trophoblast Invasion Machinery - A Functional Study *In Vitro*

**DOI:** 10.1371/journal.pone.0028514

**Published:** 2011-12-08

**Authors:** Nikola Kolundžić, Žanka Bojić-Trbojević, Tamara Kovačević, Ivana Stefanoska, Toshihiko Kadoya, Ljiljana Vićovac

**Affiliations:** 1 Laboratory for Biology of Reproduction, Institute INEP, University of Belgrade, Belgrade, Serbia; 2 Department of Biotechnology, Maebashi Institute of Technology, Maebashi, Gunma, Japan; Faculdade de Medicina, Universidade de São Paulo, Brazil

## Abstract

**Background:**

Interactions of glycoconjugates with endogenous galectins, have been long proposed to participate in several reproductive processes including implantation. In human placenta gal-1, gal-3, gal-8, and gal-13 proteins are known to be present. Each of them has been proposed to play multiple functions, but so far no clear picture has emerged. We hypothesized that gal-1 participates in trophoblast invasion, and conducted Matrigel invasion assay using isolated cytotrophoblast from first trimester placenta and HTR-8/SVneo cell line to test it.

**Methods and Findings:**

Function blocking anti-gal-1 antibody was employed to assess participation of endogenous gal-1 in cell adhesion, cell invasion of HTR-8/SVneo cells. When gal-1 was blocked in isolated trophoblast cell invasion was reduced to 75% of control (SEM±6.3, *P*<0.001) and to 66% of control (SEM±1.7, *P*<0.001) in HTR-8/SVneo cell line. Increased availability of gal-1, as two molecular forms of recombinant human gal-1 (CS-gal-1 and Ox-gal-1), resulted in increased cell invasion by cytotrophoblast to 151% (SEM±16, *P*<0.01) with 1 ng/ml of CS-gal-1, and to 192% (SEM±51, *P*<0.05) with 1 µg/ml of Ox-gal-1. Stimulation was also observed in HTR-8/SVneo cells, to 317% (SEM±58, *P*<0.001) by CS-gal-1, and to 200% (SEM±24, *P*<0.001) by Ox-gal-1 at 1 µg/ml. Both sets of results confirmed involvement of gal-1 in trophoblast invasion. Galectin profile of isolated cytotrophoblast and HTR-8/SVneo cells was established using RT-PCR and real-time PCR and found to consist of gal-1, gal-3 and gal-8 for both cell types. Only gal-1 was located at the trophoblast cell membrane, as determined by FACS analysis, which is consistent with the results of the functional tests.

**Conclusion and Significance:**

These findings qualify gal-1 as a member of human trophoblast cell invasion machinery.

## Introduction

Essential steps in achieving a successful pregnancy include development of an implantation competent blastocyst, subsequent implantation into an adequately prepared endometrium and formation of a functional placenta. During early embryo implantation and development of the human placenta, specialized trophoblast cells orchestrate a complex series of stage-specific adhesive interactions with multiple components. This has been shown in particular for invasive cytotrophoblast cells, which form the anchoring villi, attaching the conceptus to maternal decidua. As they differentiate, the invasive trophoblast cells undergo a well documented integrin switch [1] that enables invasion as well as cell adhesion in maternal interstitial and endovascular compartments [2]. The invasive trophoblast cells acquire a full repertoire of adhesion and other molecules that allow them to detach from the cell columns of the anchoring villi and invade maternal decidua. Accumulated data have shown that interactions of cell receptors with the extracellular matrix (ECM) are of particular importance for this process. The extravillous cytotrophoblast at the fetomaternal interface has been shown to produce a specific extracellular matrix that includes heavily glycosylated ECM proteins such as laminin [3] and oncofetal fibronectin [4].

Galectin-1 (gal-1) belongs to the phylogenetically conserved galectin family with an affinity for Gal-GlcNAc residues of glycoproteins. It exists as a noncovalent homodimer composed of two carbohydrate recognition domains which recognize a wide range of glycoconjugates [5], [6]. Although gal-1 has characteristics of a typical cytoplasmic protein, it can be secreted and found on the cell surface, as well as in the extracellular matrix [7], [8]. It could therefore act both inside cells, via sugar-independent interactions [9], and outside cells displaying lectin activity [10], [11]. Through recognition and interactions with β-galactoside containing ligands/counter receptors on the cell surface or in the ECM, this lectin plays a role in a variety of biological functions, including cell adhesion, migration, invasion, metastasis, apoptosis, and assembly and remodeling of ECM [7], [12]. It has been documented that gal-1 binds to various ECM components - laminin, cellular fibronectin, thrombospondin, vitronectin, ostepontin, in a dose- and β-galactoside-dependent manner [13]–[15].

The presence of gal-1 has been shown in human, as well as in mouse female reproductive tracts. This protein was the first galectin isolated, purified and cloned from human placenta [16]. It is expressed at the maternofetal interface [17]. Among placental trophoblast cells, gal-1 was immunolocalized in the cytotrophoblast of middle and distal cell columns [18] differentiating towards fully invasive trophoblast. Gal-1 is also abundantly expressed in endometrium, as well as in the decidua of early gestation [19], [20]. Other members of the galectin family, gal-3, gal-8, and gal-13, were also reported in human placenta [18], [21]–[23]. In mice [24], [25] gal-1 is synthesized prior to implantation in the trophectoderm of the expanded blastocysts, suggesting a role in the attachment of the embryo to the uterine epithelium [24]. Although studies of gal-1 and gal-3 double knockouts demonstrate no effect on reproductive phenotype, and that both molecules are dispensable for survival and fertilization, some subtle defects have been found. Therefore, it has been proposed that gal-1 and gal-3 may be molecules required for the most efficient, but not for absolute function [26], or that gal-5, also expressed by mouse blastocyst, could be involved as well [27]. However, a recent study reported that gal-1 deficient mice have higher rates of fetal loss compared to wild type mice [28], which is preventable by treatment with recombinant gal-1, suggesting its important role in the maintenance of pregnancy.

It is widely accepted that implantation of the human embryo is a process that depends on invasive properties of the trophoblast, for which both glycocode and glycan-binding proteins may play important roles [29]–[31]. Galectins bind and cross-link N-glycans on cell surface glycoproteins to form a heterogeneous lattice [32]. Gal-1 has been implicated in some of the pathological conditions involving trophoblast, such as early pregnancy loss, preeclampsia and trophoblastic malignant disease [33]–[35]. It was previously demonstrated that lactose, an inhibitory sugar for the lectin-type interaction of galectins, reduced invasion by extravillous trophoblast HTR-8/SVneo cell line [36]. In tumor biology gal-1 was shown to influence cell invasiveness [37], [38]. No direct data, however, are yet available regarding its involvement in trophoblast invasion. This study established the galectin profile of invasive trophoblast and for the first time investigated gal-1 involvement in the processes relevant for human embryo implantation, such as cell invasion, using functional tests *in vitro*.

## Materials and Methods

### Reagents and antibodies

RPMI 1640 with or without phenol red, DMEM/F12 medium, antibiotic/antimycotic solution and fetal calf serum (FCS) were obtained from PAA Laboratories (Linz, Austria). Matrigel, collagen type I and fibronectin were purchased from BD Biosciences (Bedford, MA, USA). Propidium iodide (PI), Trypan blue, acrylamide, N, N′-methylene-bis-acrylamide, N, N, N′, N′-tetramethylenediamine, Ponceau S, glycine, protease inhibitor cocktail, and MTT were obtained from Sigma Chemical Company (USA). SDS-PAGE protein standards were from Bio-Rad. The oxidized form of recombinant human gal-1 (Ox-gal-1) and a mutant form of gal-1 in which all six cysteine residues were replaced by serine (CS-gal-1) were obtained from Kirin Brewery (Japan). The following antibodies were used: rabbit anti-gal-1 with gal-1 neutralizing activity (Kirin Brewery, Japan), rabbit anti-β-actin (Sigma), mouse anti-cytokeratin-7 (CK-7, Dako, Glostrup, Denmark), rabbit anti-Ki-67 (SantaCruz Biotechniology Inc, USA), goat anti-gal-8 (R&D, Abingdon, UK) and mouse anti-gal-3 (gift from Dr M Huflejt). Non-immune rabbit IgG, non-immune mouse IgG, biotinylated horse anti-mouse, goat anti-rabbit and rabbit anti-goat IgG, avidin-biotinylated peroxidase complex (ABC) and diaminobenzidine (DAB) substrate kit for peroxidase were obtained from Vector Laboratories (Burlingame, CA, USA). Anti-mouse IgG antibody Alexa Fluor 488, anti-rabbit IgG antibody Alexa Fluor 555, anti-goat antibody IgG Alexa Fluor 488 and Prolong Gold antifade reagent with DAPI were from Molecular Probes (Invitrogen,.USA). Tris and 2-mercaptoethanol were from ICN Biomedicals, Inc. (Aurora, OH, USA). Protran nitrocellulose transfer membrane was obtained from Schleicher&Schuell BioScience GmbH Whatman Group (Dassel, Germany). TRIreagent, primers, dNTPs, AmpliTaq Gold DNA polymerase were from Applied Biosystems (Carlsbad, USA). Hexamer primers, RevertAid reverse transcriptase were obtained from Fermentas (Lithuania) and KAPA™SYBR®FAST qPCR Universal Master Mix was from Kappa Biosystems (Boston, USA). All other reagents were of the best commercial grade available.

Antibody dilutions for immunocytochemistry were 1∶75 for anti-CK7, 1∶100 for anti-gal-1, 1∶50 for Ki-67, 1∶200 for biotinylated horse anti-mouse and goat anti-rabbit IgG, and 1∶1000 for Alexa Fluor 488 anti-mouse IgG antibody and Alexa Fluor 555 anti-rabbit IgG antibody. For Western blot analysis dilutions were 1∶2500 for anti-gal-1, 1∶10 for anti-gal-3, 1∶500 for anti-gal-8 for primary cytotrophoblast (CT) and HTR-8/SVneo cells, 1∶500 for anti-actin (HTR-8/SVneo cells) and 1∶1000 for primary CT. Biotinylated horse anti-mouse IgG was diluted 1∶750 for HTR-8/SVneo cells and 1∶1000 for isolated trophoblast cells, goat anti-rabbit IgG was diluted 1∶1200 for both cell types and rabbit anti-goat was diluted 1∶600 (HTR-8/SVneo) and 1∶800 for primary CT in Western blot analysis.

### Isolation of CT and cell culture

HTR-8/SVneo cells were kindly provided by Dr Charles H Graham (Queen's University, Kingston, Ontario, Canada). This cell line was established from human first trimester explant cultures immortalized by SV40 large T antigen [39], [40]. Cytotrophoblast cells were isolated from the first trimester of pregnancy placentas from legal abortions (6–12 weeks) undertaken for non-medical reasons. Placental tissues were obtained from subjects undergoing voluntary termination of pregnancy at Clinic for Obstetrics and Gynecology, Clinical Center of Serbia, Belgrade, or Military Medical Academy, Belgrade, after a verbal consent was given. This was in keeping with the agreements between each of these institutions and INEP, and according to local ethical standards (document GSP/05; PR030/09) approved by Institutional committee of Institute for the Application of Nuclear Energy, INEP.

The isolation protocol using sequential trypsin digestion has been reported previously [41], [42]. After isolation, cells were identified as trophoblast by immunocytochemical staining for CK-7, and preparations with >90% cytokeratin positive cell were used for all experiments. Since cell preparations also contained a small proportion of CD45-positive cells (<6%), these cells were removed using immunomagnetic beads prior to Western blot, FACS analysis, and RNA isolation, as described below.

Cytotrophoblast was cultured in DMEM/F12 supplemented with 10% FCS (v/v), and HTR-8/SVneo cells in RPMI 1640 supplemented with 5%FCS (v/v), and with antibiotic/antimycotic solution. For SDS-PAGE, isolated CT cells or trypsinized HTR-8/SVneo cells were washed in 0.05 M phosphate buffered saline (PBS), pH 7.2, lysed in sample buffer containing protease inhibitor cocktail (6×10^6^ cells/ml), centrifuged (1600 g for 5 min at 4°C) and the supernatant reserved. Cell lysates were heated for 5 min in boiling water bath and subjected to SDS-PAGE.

For immunocytochemical analysis CT and HTR-8/SVneo cells were cultured on glass cover slips in their respective media at 37°C in a moist atmosphere of air with 5% CO_2_. Isolated CT was cultured for 18–20 h. Subconfluent HTR-8/SVneo cells were also cultured with non-immune rabbit IgG or anti-gal-1 (1 µg/ml) for 24 h at 37°C. Cover slips were then rinsed twice with PBS and fixed with ice-cold acetone-methanol (1∶1) for 10 min. Cover slips were kept frozen until stained.

For PI and Ki-67 staining, and MTT test, HTR-8/SVneo cells were cultured in 96-well plates at 1×10^5^, for FACS analysis, and 2×10^4^ cells/well for MTT, in 100 µl of complete RPMI 1640. After 24 h, HTR-8/SVneo cells were briefly rinsed with sterile PBS (sPBS) and further cultured in control or one of the treatment media containing: a) anti-gal-1 (1 and 5 µg/ml), b) non-immune rabbit IgG (1 and 5 µg/ml), c) CS-gal-1 (1 ng/ml, 0.1 and 1 µg/ml), d) Ox-gal-1 (1 ng/ml, 0.1 and 1 µg/ml), dissolved in RPMI 1640 without phenol red containing 0.1% bovine serum albumin (BSA), to a total culture volume of 200 µl. After 24 h, the cells were rinsed twice with warm sPBS.

For RNA isolation, total RNA was collected from subconfluent cultures of HTR-8/SVneo and isolated cytotrophoblast cells using TRIreagent according to the manufacturer's instructions.

### Immunocytochemistry

Cytotrophoblast from primary culture and HTR-8/SVneo cells on coverslips were washed with PBS, air-dried and fixed with ice-cold acetone-methanol. Endogenous peroxidase activity was blocked with 1% hydrogen peroxide for 30 min, and non-specific binding of proteins with 1% casein in PBS for 20 min at room temperature (RT). Cells were then incubated with anti-CK-7 antibody for 1 h at RT, followed by incubation with biotinylated horse anti-mouse IgG for 30 min and with ABC for another 30 min. The reaction was visualized using DAB as chromogen. In double immunostaining experiments, cytokeratin stained trophoblast cells were subsequently incubated with anti-gal-1 antibody overnight and visualized using anti-rabbit IgG Alexa Fluor 555 antibody. Slides were mounted with Prolong Gold antifade reagent with DAPI and examined using a Carl Zeiss Axio Imager 1.0 microscope with AxioCam HR monochrome camera (Jena, Germany), or with a Canon A640 Digital Camera System (Tokyo, Japan).

Negative controls were performed routinely. Omission of primary antibody and use of non-immune serum in place of specific antibody resulted in complete absence of staining. HTR-8/SVneo cells cultured with non-immune rabbit IgG or anti-gal-1 were incubated with secondary anti-rabbit IgG Alexa Fluor 555 antibody only.

### Immunohistochemistry

Immunohistochemistry was performed on paraffin-embedded placental tissue from first trimester of pregnancy. Endogenous peroxidase activity was quenched by 30 min incubation in 1% (v/v) H_2_O_2_/ethanol followed by a wash with distilled water. Nonspecific binding was blocked by 20 min incubation with 1% casein. Sections were incubated with anti-gal-1, overnight at 4°C, and stained with anti-rabbit IgG Alexa Fluor 555 antibody. For the negative control primary antibody was omitted.

### Cell adhesion

Adhesion assays were performed in 96-well plates in wells either uncoated or precoated with 250 ng/well of fibronectin, Matrigel or collagen type I for 1 h at 37°C. The plates were treated with 100 µl 3% BSA in PBS for 30 min at 37°C to block non-specific binding sites, washed with PBS, and used in the adhesion assay. The HTR-8/SVneo cells were detached with 5 mM EDTA in PBS and resuspended at a final concentration of 2.5×10^5^/ml in 0.1% BSA-RPMI 1640 without phenol red. Cells were preincubated with or without 0.1 M lactose, non-immune rabbit IgG or anti-gal-1 (1 and 5 µg/ml) for 1 h in a humidified atmosphere of 5% CO_2_ at 37°C with occasional agitation, and plated at 2.5×10^4^ cells/well. After incubation for 1 or 2 h, the unattached cells were removed, and the plates were gently rinsed once with PBS, and fixed with ice-cold acetone/methanol (1∶1) for 10 min. The attached cells were stained by adding 0.05% crystal violet in 25% methanol at 50 µl/well for 5 min. The excess of dye was removed by immersing the plates in water and drying at RT. The incorporated dye was dissolved in 0.1 M sodium citrate in 50% ethanol at 100 µl/well and optical density was read at 540 nm. The intensity of staining was proportional to the number of adhered cells. The experiments were repeated three times.

### HTR-8/SVneo cell survival in the presence of gal-1

Cell viability was assessed by MTT assay [43]. HTR-8/SVneo cells were cultured in 96-well plates as described above. After treatment, 100 µl of MTT (2.4 mmol/l) in 10% FCS/PBS (v/v) were added to each well. After incubation for 2 h at 37°C, the medium was replaced by 1-propanol (100 µl/well), and the plates were vigorously shaken to ensure complete solubilizaiton of the blue formazan. Absorbance was measured at 570 nm using a microplate reader and the cells were quantified using the standard curve obtained with 5×10^3^, 1×10^4^, 2×10^4^, 4×10^4^, 6×10^4^, or 8×10^4^ cells/well. The experiment was repeated four times for both concentrations of anti-gal-1 antibodies, non-immune rabbit IgG and 0.1 M lactose, or three times for Ox-gal-1 and CS-gal-1 (1 ng/ml, 0.1 and 1 µg/ml).

The effect of gal-1 on cell proliferation was investigated by immunostaining of HTR-8/SVneo cells for Ki-67 nuclear antigen followed by FACS analysis, as detailed bellow. Presence of apoptotic HTR-8/SVneo cells was determined by PI staining after incubation with Ox-gal-1 and CS-gal-1 (1 ng/ml and 1 µg/ml). For PI, cells were washed with cold PBS and resuspended in 300 µl of hypotonic solution (0.1% sodium citrate and 0.1% Triton X-100 in distilled water) and PI (40 µg/ml), and kept at 4°C overnight, before quantitation by EPICS XL-MCL flow cytometer.

### Cell invasion assay

The transwell invasion assay was conducted in 24 well plates with membrane inserts (8 µm pore size; Millipore), as described previously [44], with minor modifications. Insert membranes were pre-coated with 10 µl of growth factor-reduced Matrigel at 2 mg/ml for 30 min at 37°C. Isolated CT (2×10^5^/200 µl) and HTR-8/SVneo cells (1×10^5^/200 µl) were resuspended in one of the treatment media with 0.1% BSA containing: anti-gal-1 or non-immune IgG (1 and 5 µg/ml), 0.1 M lactose, CS-gal-1 or Ox-gal-1 (1 ng/ml and 1 µg/ml for primary CT; 1 µg/ml for HTR-8/SVneo), 0.1 M lactose with 1 µg/ml CS-gal-1 or Ox-gal-1 (HTR-8/SVneo). For control, cells were resuspended in medium without treatment. Cells were then seeded in the upper chamber of Matrigel-coated transwells. The lower chambers were loaded with 500 µl of medium without or with anti-gal-1 or non-immune IgG, 0.1 M lactose, CS-gal-1 or Ox-gal-1, and 0.1 M lactose and CS-gal-1 or Ox-gal-1. After incubation for 24 h in a humidified atmosphere, filters were washed twice in warm PBS, and cells on the upper surface were gently removed with a cotton swab. Cells were fixed with ice-cold acetone-methanol (1∶1) for 10 min. Filters were stained using primary anti-CK-7 for primary trophoblast by the same procedure as described above, and by Giemsa staining for HTR-8/SVneo cells. Cells on the underside of the filters and the pores occupied were counted in non-overlapping fields of the whole membranes under a light microscope (Reichert-Jung with Leica DC150 Digital Camera System). The CT cells used were isolated from nine placentas for treatment with anti-gal-1 and lactose, from four placentas for CS-gal-1 treatment and from five placentas for Ox-gal-1 treatment. The experiments were repeated four times in duplicate for 1 µg/ml of anti-gal-1; eight times in duplicate for 5 µg/ml of anti-gal-1; three times in duplicate for each molecular form of gal-1 for HTR-8/SVneo cells. Data presented for HTR-8/SVneo cells were expressed as a percentage of the control values. In the Matrigel invasion test, isolated CT differentiates to non-proliferative trophoblast.

### RT-PCR

First-strand cDNA was synthesized from 2 µg of total RNA, using 0.2 µg of random hexamer primers (Fermentas, Lithuania), 2.5 µM of each dNTP (Applied Biosystems) and 200 U of RevertAid reverse transcriptase (Fermentas, Lithuania) in a total volume of 20 µl, according to the manufacturer's instructions. Each gene was amplified by PCR reaction in a final volume of 25 µl containing 200 ng of cDNA (2 µl of RT mixture), 2.5 µM of each dNTP, 1 µM of each of the primers (sequences previously published in 45) and 0.25 U of AmpliTaq Gold DNA polymerase. The temperature profile included initial denaturation at 95°C for 10 min followed by 35 cycles of 1 min at 95°C, 1 min at 60°C, and 1 min at 72°C, and a final extension step at 72°C for 10 min. PCR products were analyzed electrophoretically on 2% agarose gel and visualized by ethidium bromide staining and UV light.

### Real-time PCR

Quantitative real-time PCR was performed using the SYBR®Green chemistry in a 7500 Real Time PCR System (Applied Biosystems, Carlsberg, USA). The reaction mixture in a final volume of 10 µl contained 100 ng of cDNA (1 µl of RT mixture), 5 µl 2× KAPA™SYBR®FAST qPCR Universal Master Mix and gene-specific primers [45] in final concentration of 0.4 µM. The cycling conditions were as follows: denaturation step at 95°C for 10 min followed by 40 cycles of 95°C for 15 s and 60°C for 1 min. Each sample was amplified in duplicate, followed by melting curve analyses to verify amplification specificity. All primers exhibited optimal amplification efficiencies in serial dilution experiments. GAPDH was used as the endogenous control gene. Calculations were made using the comparative ddCt method [46].

### SDS-PAGE and immunoblotting

SDS-PAGE was performed on 15% polyacrylamide gel and 4% stacking gel under reducing conditions. All samples (HTR-8/SVneo and CT cell lysates) were prepared by boiling in 0.125 M Tris-HCl buffer, containing 4% SDS (w/v), 20% glycerol (v/v), 0.1% bromphenol blue, and 10% 2-mercaptoethanol (v/v) for 5 min, and 80 µg of protein were loaded per lane. Bio-Rad molecular weight markers were used according to the manufacturers' instructions. The proteins separated by electrophoresis were tranferred onto nitrocellulose membranes. Transfer was performed at constant voltage (15 V) for 30 min, and was confirmed by Ponceau S staining of the membranes. Non-specific binding sites on membranes were blocked with 5% casein in PBS (w/v), at RT for 1 h. After blocking, membranes with immobilized antigens were incubated with: rabbit anti-gal-1, mouse anti-gal-3 and goat anti-gal-8 overnight at 4°C, with constant shaking. Subsequent to five washes for 5 min each, blotting membranes were incubated with the corresponding biotinylated secondary antibody for 30 min. After intensive washing, membranes were incubated with ABC for 30 min. Bound conjugates were visualized using DAB/Ni as chromogen. Non-specific binding was estimated by omitting the specific antibody. Staining for actin was used as the loading control. Membranes were scanned and analyzed by the ImageMaster TotalLab v2.01 program (Amersham Biosciences).

### Flow cytometry

Flow cytometry was used to analyse expression of intra- and extracellular gal-1, gal-3 and gal-8 on HTR-8/SVneo and isolated trophoblast cells. Magnetic beads coated with antibody to CD45 were employed to deplete bone-marrow derived cells from the isolated CT cell suspension. Dynabeads – M-280 sheep anti-mouse IgG (Invitrogen Dynal AS, Oslo, Norway) were coated with mouse anti-CD45 MAB (1∶100; Serotec, Oxford, UK) according to the manufacturer's instructions. The coated beads were stored in PBS/0.1% BSA at 4°C until use. Isolated cell suspensions were incubated with coated beads for 1 h at 4°C with rotation. Beads with attached CD45-positive cells were removed magnetically. The resulting CT cells were washed four times with PBS, and the purity was estimated by cytospins stained with anti-CD45 and anti-CK-7 antibodies. The purity of stained cells was >98%. Purified CT cells and HTR-8/SVneo cells detached with cold 5 mM EDTA were washed twice with cold PBS_2_ (PBS, 2% FCS, and 0.01% sodium azide), and were permeabilized with fixation/permeabilization concentrate diluted in fixation/permeabilization diluent, 1∶4 (eBioscience, San Diego, CA, USA), overnight at 4°C. Cells were washed twice in permeabilization buffer (PB), diluted in deionized water (1∶10), and incubated with rabbit anti-gal-1, mouse anti-gal-3, goat anti-gal-8, and rabbit anti-Ki-67 antibodies for 45 min at 4°C. Non-permeabilized cells were incubated with the same antibodies. After incubation and subsequent washing with PB, cells were stained with anti-rabbit IgG Alexa Fluor 555 antibody (gal-1), anti-mouse IgG Alexa Fluor 488 (gal-3) and anti-goat Alexa Fluor 488 (gal-8). Control cells were incubated with non-immune rabbit IgG, non-immune mouse IgG and non-immune goat IgG as the primary antibodies. Labeled cells were fixed with 4% formalin, and were analyzed on an EPICS XL-MCL flow cytometer (Coulter, Krefeld, Germany).

### Statistical analysis

The data were analyzed with the Statistical Software Program, version 5.0 (Primer of Biostatistic, McGraw-Hill Companies, Inc., New York, NY, USA) using the one-way analysis of variance (ANOVA). Values were considered significantly different when *P*<0.05.

## Results

**Figure 1:**
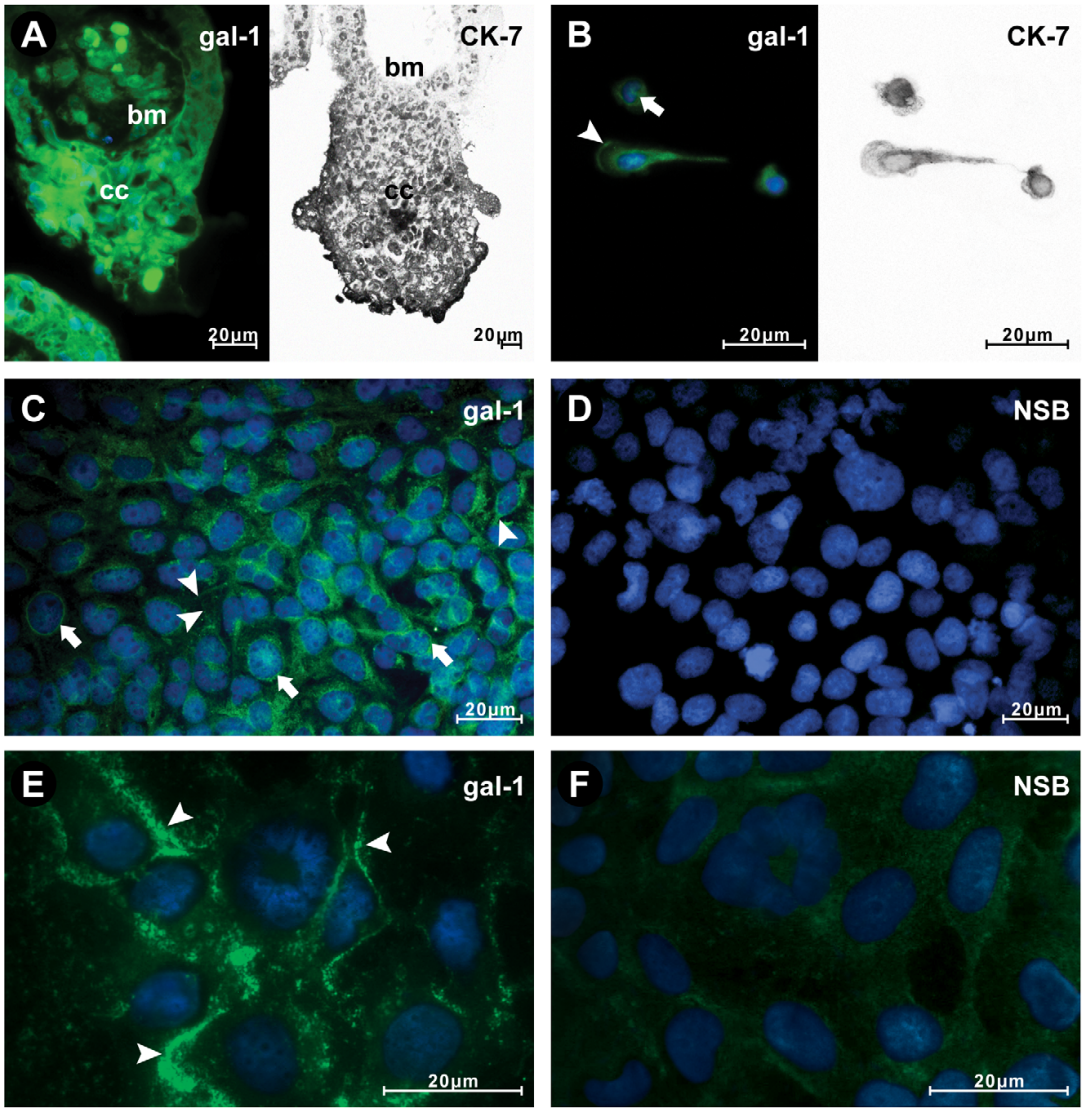
Anti-gal-1 (used throughout the study to block trophoblast cell function *in vitro*) localized gal-1 (A left) to extravillous CT of the trophoblast cell column in tissue sections (CC), isolated cytotrophoblast in primary culture (B left), HTR-8/SVneo extravillous cell line (C), and bound to HTR-8/SVneo cells in culture (E). Trophoblast identity of isolated trophoblast cells (B right) and cell column (A right) was confirmed by staining for CK-7. The negative control for immunocytochemistry is shown in D. Gal-1 is present within cells (arrows in B, C) and associated with the cell membrane (arrowheads in A, B, C, E; BM is basement membrane in A). For gal-1 function-blocking experiments HTR-8/SVneo cells were cultured with either anti-gal-1 or non-immune rabbit IgG for 24 h. Binding was detected in fixed monolayers by secondary antibody (as detailed in Material and Methods), and shown in E as membrane staining for gal-1 (arrow heads) and in F for non-specific binding (NSB). Cells were counterstained with DAPI to visualize the nuclei (blue).

Expression of gal-1 in human placenta has been established previously [16], [17]. Among other placental cell types, the presence of gal-1 has been documented for extravillous cytotrophoblast [18], [19], and also for some trophoblast derived cell lines [18], [36]. This study aimed at investigating the possibility that gal-1 participates in processes relevant for human trophoblast invasion, using anti-gal-1 with demonstrated function-blocking activity. Therefore, our preliminary task was to establish binding of this particular anti-gal-1 in placental tissue, fixed cells and during cell culture. The anti-gal-1 was found to bind to gal-1 in placental tissue (Fig. 1A) and both trophoblast cell types used in this study, HTR-8/SVneo (Fig. 1C, E) and isolated cytotrophoblast in primary culture (Fig. 1B). The identity of cell column trophoblast and isolated cytotrophoblast was confirmed by anti-cytokeratin-7 staining in Fig. 1A right, and 1B right, respectively). In trophoblast cell columns (CC) and both cell types gal-1 was localized within cells, often perinuclearly (arrows in Fig. 1B and C), as well as in the cell membrane domain or extracellularly (arrowheads Fig. 1B and C). The functional tests employed here were based on the use of an anti-gal-1 with previously demonstrated function-blocking activity [47]. Binding of this antibody to the cell membrane of the HTR-8/SVneo cells in culture is shown (arrowheads in Fig. 1E), which was not the case with non-immune rabbit IgG (Fig. 1F). This finding confirmed that this antibody specifically recognized extravillous trophoblast gal-1 and bound to cultured cells, making it suitable for further use in the functional studies shown below.

### Cell adhesion of HTR-8/SVneo cells is influenced by lectin type interactions and availability of gal-1

**Figure 2:**
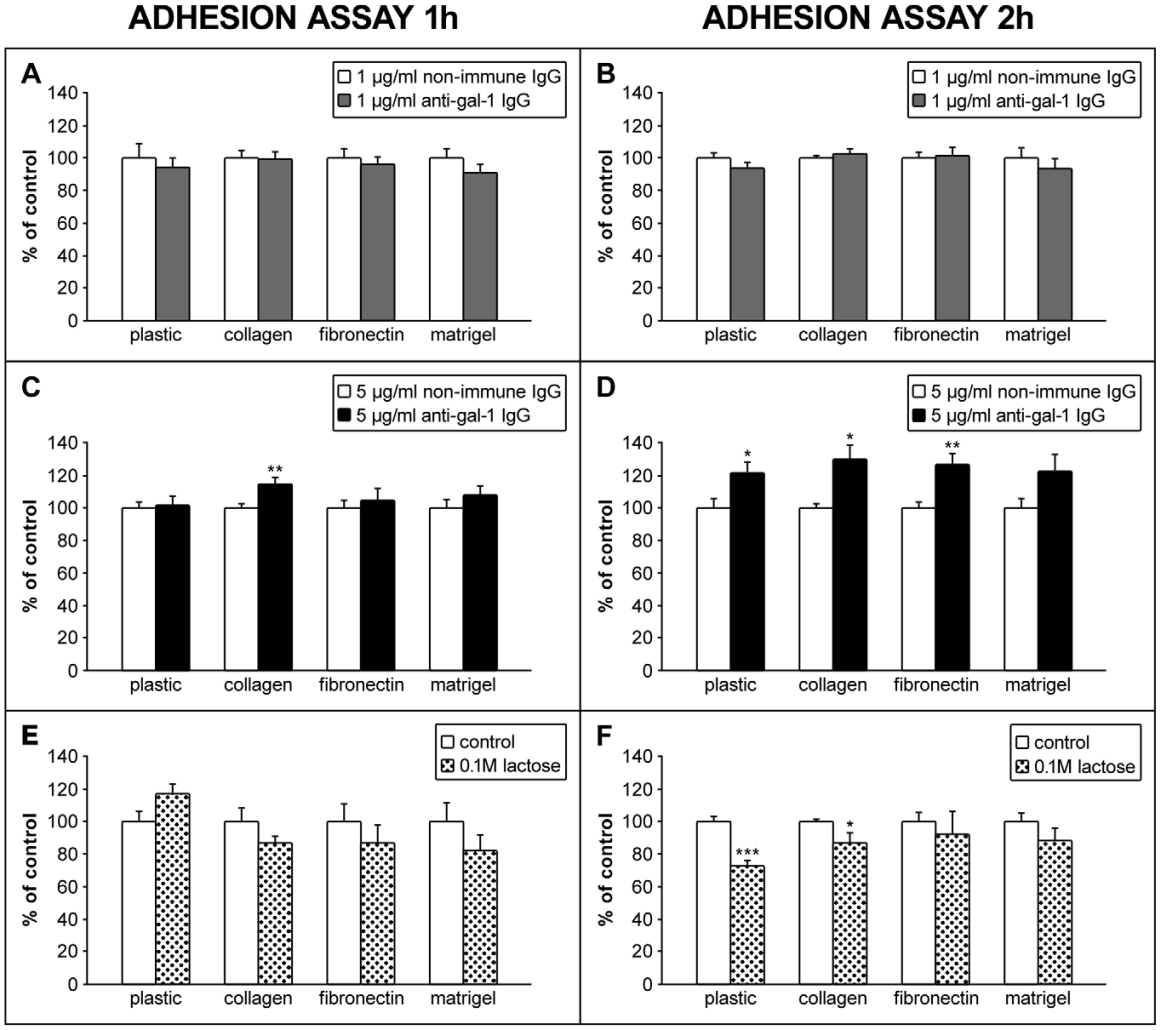
Effect of function blocking anti-gal-1 antibodies and lactose on adhesion of HTR-8/SVneo cells to plastic, collagen type I, fibronectin and Matrigel. Adhesion was studied after 1 h (A, C) or 2 h (B, D) using anti-gal-1 antibody (1 µg/ml in A and B; 5 µg/ml in C and D) in adhesion to uncoated or precoated wells. The relevance of lectin-type interactions for extravillous trophoblast cell adhesion was assessed in the presence of 0.1 M lactose after 1 h and 2 h (E, F). Data (from three experiments in triplicate) are expressed as percentages of non-immune IgG or untreated controls, with values given as mean ± S.E.M., and statistical significance as * for *P*<0.05, ** for *P*<0.01 and *** for *P*<0.001.

Adhesion of HTR-8/SVneo cells pretreated with non-immune IgG or function-blocking antibody (1 or 5 µg/ml) was studied at two time points 1 h (Fig. 2A, C) and 2 h (Fig. 2B, D), using plastic or ECM protein coated surfaces. In order to assess participation of lectin-type interactions in HTR-8/SVneo cell adhesion to these surfaces, cells were also pretreated and incubated in the absence (control) or presence of 0.1 M lactose (Fig. 2E, F). When the cells were cultured on plastic or surfaces coated with collagen type I, fibronectin, or Matrigel, no difference in cell adhesion was observed with the anti-gal-1 antibody at 1 µg/ml compared to the control after either time interval. With an anti-gal-1 concentration of 5 µg/ml, however, after 1 h there was a small increase in adhesion on collagen type I (115%, *P*<0.01). After 2 h of culture under the same conditions adhesion to all surfaces was increased: to 122% (*P*<0.05) on plastic, to 130% on collagen I (*P*<0.01), to 127% on fibronectin (*P*<0.01) and to 123% on Matrigel (not significant). Dependance on anti-gal-1 concentration suggested involvement of gal-1 in adhesion, possibly through its lectin activity, which was further tested in experiments with 0.1 M lactose, as an inhibiting sugar for galectins. Cells were either pretreated with 0.1 M lactose or kept in control medium for 1 h before plating on different ECM coated surfaces and were further cultured under the same conditions for 1 or 2 h. After 1 h of culture there was no difference compared to the control on any of the studied surfaces (Fig. 2E), but after 2 h of incubation the presence of 0.1 M lactose significantly reduced adhesion to plastic to 72% of the control (*P*<0.001), and to a lesser degree to collagen I (87% of the control, *P*<0.05). This finding suggests that lectin-type interactions may contribute to cell adhesion of HTR-8/SVneo cells, but the nature of the interactions remained obscure at this point.

### Galectin-1 and HTR-8/SVneo cell survival

**Figure 3:**
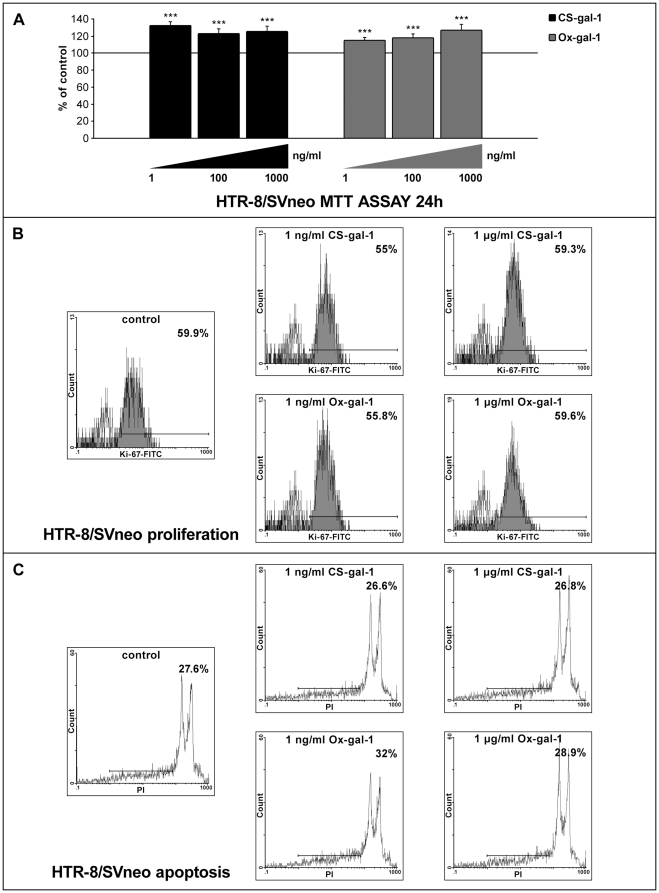
Gal-1 influence on HTR-8/SVneo cell survival. The effect of mutant gal-1, CS-gal-1 (A left) and oxidized gal-1, Ox-gal-1 (A right) on HTR-8/SVneo cell viability. Cells were maintained for 24 h in RPMI 1640 medium with 0.1% BSA without (control) or with CS-gal-1 or Ox-gal-1 (1, 100 and 1000 ng/ml). Values are given as percentages of control values (mean ± S.E.M.), three experiments, six replicates each. Differences *vs* control significant at *P*<0.001 (***). Effect of the same gal-1 forms (1 ng/ml and 1 µg/ml) on proliferation (B), evidenced as percent of Ki-67 positive cells, and apoptosis (C), evidenced by PI staining (FACS analysis).

Invasive extravillous trophoblast does not proliferate *in vivo*. Nevertheless, since several members of the galectin family were shown to affect cell proliferation in other models [48]–[50], this possibility was tested for gal-1 on HTR-8/SVneo cells using the MTT test of cell viability (Fig. 3). Two molecular forms of recombinant human gal-1 [47], [51] were used: stabilized gal-1 mutant (CS-gal-1, Fig. 3A left) with lectin activity (of gal-1) and oxidized gal-1 (Ox-gal-1, Fig. 3A right) without lectin activity [52]. The data obtained after 24 h indicated that both molecular forms at all concentrations studied increased (*P*<0.001) absorbance, compared to the control. CS-gal-1 (Fig. 3A left) induced maximal stimulation of 132% of the control value with 1 ng/ml, which was not further increased at higher concentrations (0.1 µg/ml and 1 µg/ml). The effect of Ox-gal-1 (Fig. 3A right) was dose dependent (1 ng/ml, 0.1 and 1 µg/ml), with maximal stimulation of 127% at 1 µg/ml. Also, when the MTT assay was performed with anti-gal-1 function-blocking antibody (1 and 5 µg/ml) no effect on HTR-8/SVneo cell viability was obtained (data not shown). Further investigation, however, demonstrated no change in HTR-8/SVneo cell proliferation (Fig. 3B) or apoptosis (Fig. 3C). Percent of proliferating cells was not altered in any culture treated with either CS-gal-1 or Ox-gal-1, compared to the control of 55–60% positive cells. Similarly, the same treatments did not induce any change in apoptotic cells, being 27–32% of total cells.

### Endogenous as well as added recombinant gal-1 promote trophoblast cell invasion

**Figure 4:**
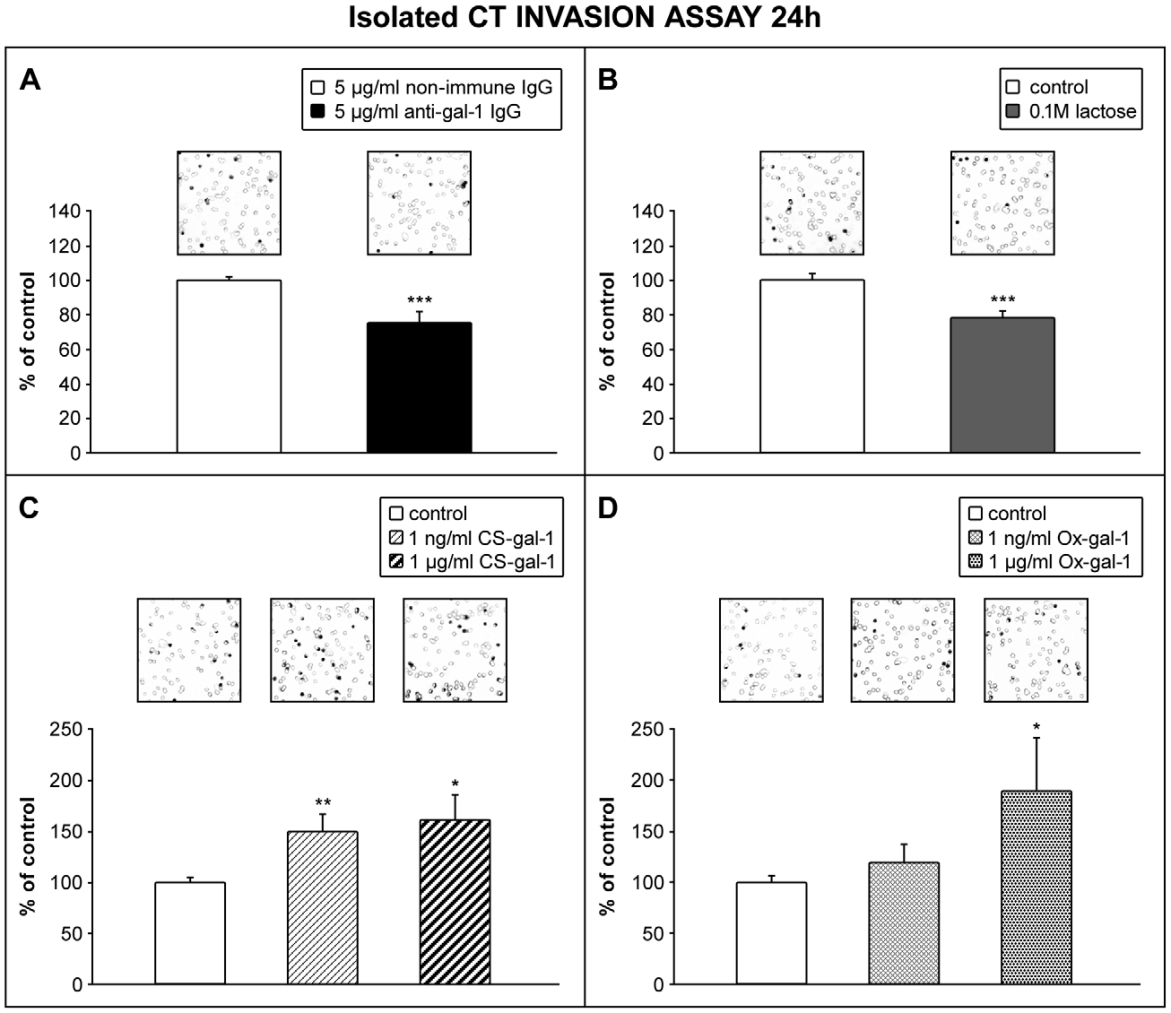
Availability of gal-1 influences cell invasion by isolated cytotrophoblast. The freshly isolated primary cytotrophoblast cells were incubated during invasion with 5 µg/ml of non-immune rabbit IgG or function-blocking anti-gal-1 antibody (A), 0.1 M lactose (B), 1 ng/ml and 1 µg/ml CS-gal-1 (C) and 1 ng/ml and 1 µg/ml Ox-gal-1 (D). Cells on the underside of the filters and the occupied pores were counted after 24 h of culture. The data are expressed as the percentage of pores occupied in the treatment compared to the control, with values given as mean ± S.E.M. Cell invasion by isolated trophoblast was reduced in the presence of function-blocking anti-gal-1 (A, *P*<0.001), or by lactose (B, *P*<0.001) as an inhibiting sugar. Treatment with CS-gal-1, 1 ng/ml and 1 µg/ml, induced stimulation of trophoblast invasion (C), *P*<0.01 and *P*<0.05 respectively, but to a lesser degree with Ox-gal-1 (D, only with 1 µg/ml, *P*<0.05). Squares above the bars represent typical fields of the invasion insert. Data from at least four individual placentas per treatment, treatments in duplicate or triplicate, entire inserts were counted.

**Figure 5:**
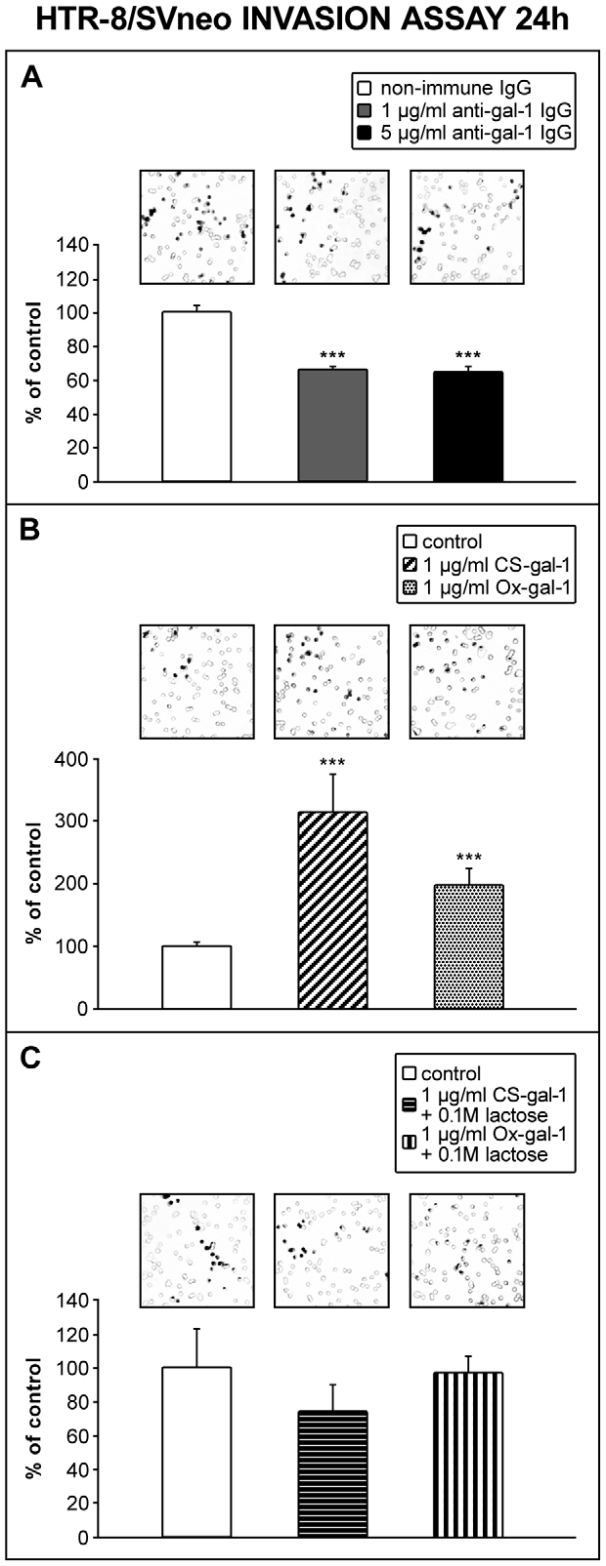
Availability of gal-1 influences HTR-8/SVneo cell invasion. Cells were incubated with 1 and 5 µg/ml of non-immune IgG or anti-gal-1 antibodies (A), 1 µg/ml of CS-gal-1 or Ox-gal-1 alone (B) or in combination with 0.1 M lactose (C). Cells on the underside of the filters and the occupied pores were counted after 24 h of culture. The data are expressed as the percentage of pores occupied in the treatment compared to the control value, with data given as mean ± S.E.M. For treatments with CS-gal-1 and Ox-gal-1 data were normalized to take into account the increase in cell number.The presence of function-blocking anti-gal-1 (A, at 1 and 5 µg/ml) significantly reduced invasion with no difference between the two concentrations (*P*<0.001, four experiments, in duplicate for 1 µg/ml; *P*<0.001, eight experiments in duplicate for 5 µg/ml of anti-gal-1 IgG). The addition of gal-1 significantly stimulated cell invasion, which was considerably lower for Ox-gal-1 than for CS-gal-1 at 1 µg/ml (B, *P*<0.001 for CS-gal-1, *P*<0.001 for Ox-gal-1; three experiments in duplicate for each molecular form of gal-1). This stimulatory activity was abolished in the presence of 0.1 M lactose for both forms of recombinant gal-1 (C). Squares above the bars represent typical fields of the invasion insert.

The focus of this study was to establish whether gal-1 is relevant for the process of trophoblast invasion. This was approached using a Matrigel invasion assay with isolated cytotrophoblast from first trimester of pregnancy placenta or HTR-8/SVneo cells, in which endogenous gal-1 was targeted by function-blocking anti-gal-1. The results obtained for primary cytotrophoblast (Fig. 4) showed that the presence of anti-gal-1 (5 µg/ml) decreased invasion to 75% of the control (*P*<0.001), where non-immune IgG was present (Fig. 4A). Therefore, endogenous gal-1 was proposed to participate in trophoblast cell invasion. Another set of experiments was conducted with a cytotrophoblast Matrigel invasion system in which the inhibiting sugar, lactose (0.1 M), was present (Fig. 4B). Our previous data revealed that HTR-8/SVneo cell invasion was reduced in the presence of lactose [34], which was confirmed here for isolated cytotrophoblast with the observed inhibition to 78% of the control value (*P*<0.001). Taken together, the data suggest that lectin-type interactions of endogenous galectins, and gal-1 in particular, participate in trophoblast cell invasion. Whether increased availability of functional gal-1 positively correlated with cell invasion *in vitro* was tested further by adding either the CS-gal-1 or Ox-gal-1 form of recombinant human gal-1 to the invasion test system (Fig. 4C, D). In the presence of as little as 1 ng/ml of CS-gal-1 Matrigel invasion by cytotrophoblast was increased to 151% of the control (*P*<0.01), but was only slighly increased further with 1 µg/ml (161% of control, *P*<0.05). However, Ox-gal-1, which has reduced or no gal-1 lectin activity under the conditions used, did not change cytotrophoblast cell invasion at 1 ng/ml, but induced stimulation to 192% with 1 µg/ml (*P*<0.05). A similar set of tests was conducted on HTR-8/SVneo cells (Fig. 5). Function-blocking anti-gal-1 reduced cell invasion to 66% of the control (*P*<0.001) with 1 µg/ml, while an increased concentration of 5 µg/ml, did not decrease invasion further (64% of the control incubated with non-immune IgG at the same concentration; Fig. 5A). Thus, blocking endogenous gal-1 was effectively induced by 1 µg/ml antibody and the effects were comparable on the two studied trophoblast cell types. Addition of mutant CS-gal-1 or recombinant Ox-gal-1 (Fig. 5B) on the other hand, stimulated HTR-8/SVneo cell invasion to 317% of the control for CS-gal-1 (1 µg/ml, *P*<0.001), and 200% for Ox-gal-1 (1 µg/ml, *P*<0.001). When lactose (0.1 M) was added to the system, stimulation of invasion with either molecular form of gal-1 was completely abolished (Fig. 5C).

### Gal-1, gal-3 and gal-8 comprise galectin profile of the invasive trophoblast

**Figure 6:**
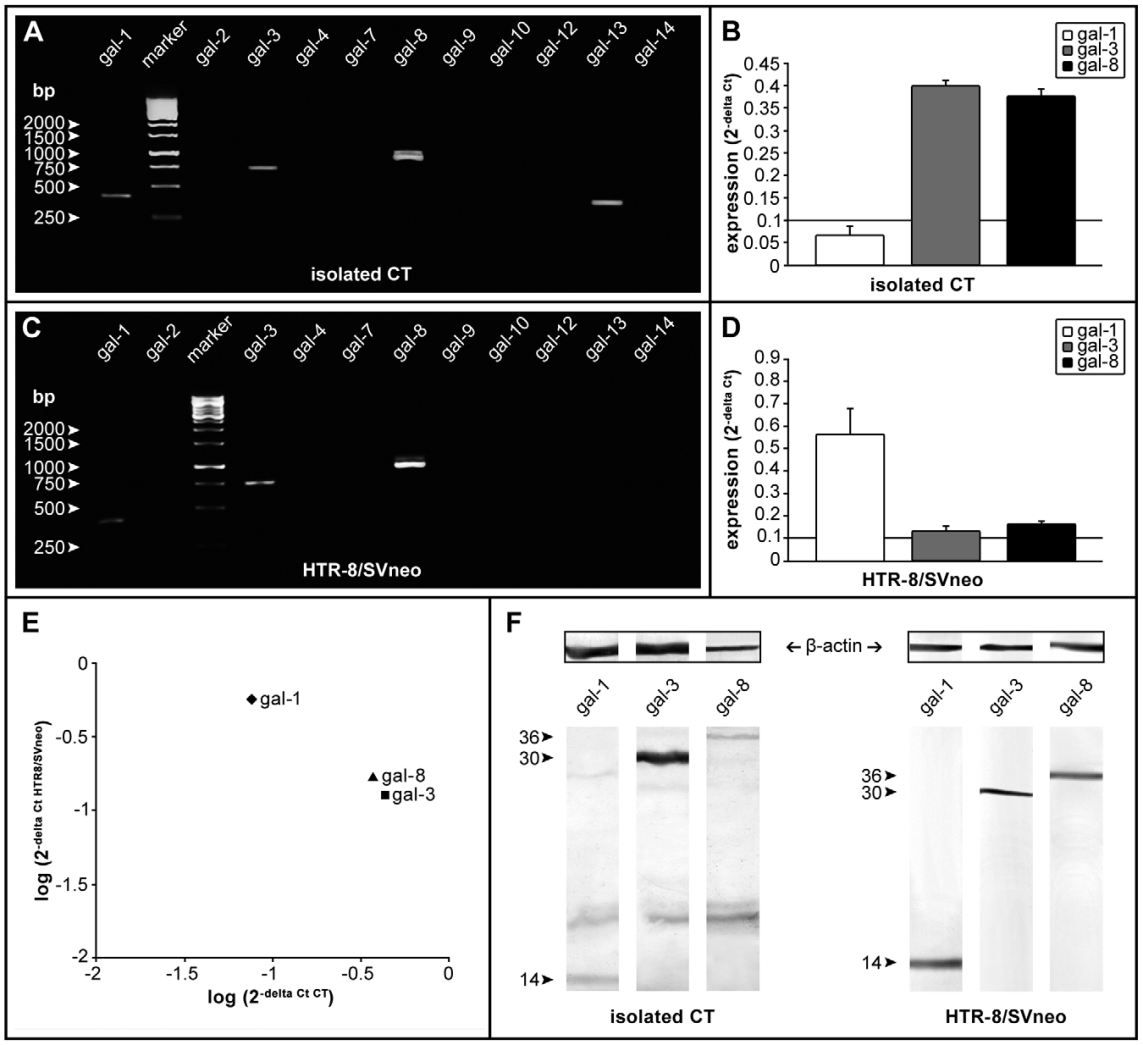
Galectin expression profile of freshly isolated trophoblast and extravillous cell line HTR-8/SVneo. RT-PCR analysis demonstrates the presence of transcripts for gal-1, gal-3 and gal-8 in both isolated cytotrophoblast (A) and HTR-8/SVneo cell line (C) and gal-13 from contaminating ST in the CT cell preparation. The sizes of standard (DNA) fragments are indicated by arrow heads. Relative levels of gal-1, -3 and -8 were determined by real-time PCR in both cell types (B for isolated CT, D for HTR-8/SVneo; internal control GAPDH). Comparison between the two cell types (E) isolated CT (*x*-axis) and extravillous cell line HTR-8/SVneo (*y*-axis). Western blot analysis of gal-1, gal-3 and gal-8 proteins in whole cell lysates of freshly isolated cytotrophoblast and HTR-8/SVneo cells (F), resolved by SDS-PAGE on 15% gel under reducing conditions. Densitometric analysis by ImageMaster TotalLab v2.01 programme. Protein bands of appropriate molecular mass are indicated with arrow heads; β-actin served as the loading control. A representative experiment of three for each cell type is shown for RT-PCR, real-time PCR and immunoblot.

**Figure 7:**
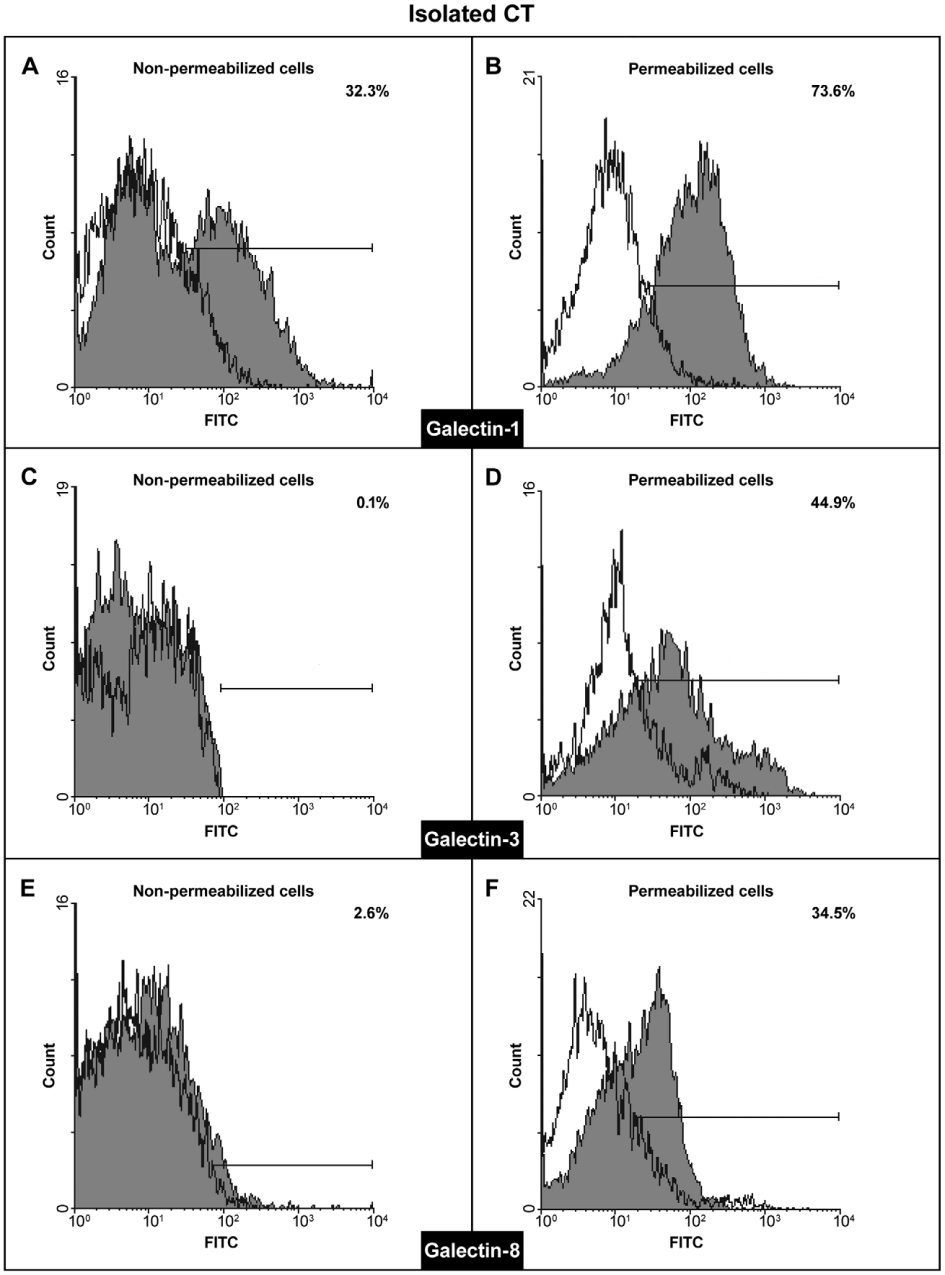
Cytofluorometrical analysis of the expressed galectin proteins in isolated CT. The *x*-axis indicates fluorescence intensity measured on a log10 scale, and the *y*-axis indicates event counts on a linear scale. Specific staining is shown as gray, and non-specific staining (isotype-matched control IgG) as white histograms. Either non-permeabilized (A, C, E), or permeabilized cells (B, D, F) were probed for expression of gal-1 (A, B), gal-3 (C, D) and gal-8 (E, F) using appropriate antibodies. The percentages of non-permeabilized and permeabilized gal-1, -3 and -8 positive cells are shown in each histogram. A representative experiment of three is shown.

**Figure 8:**
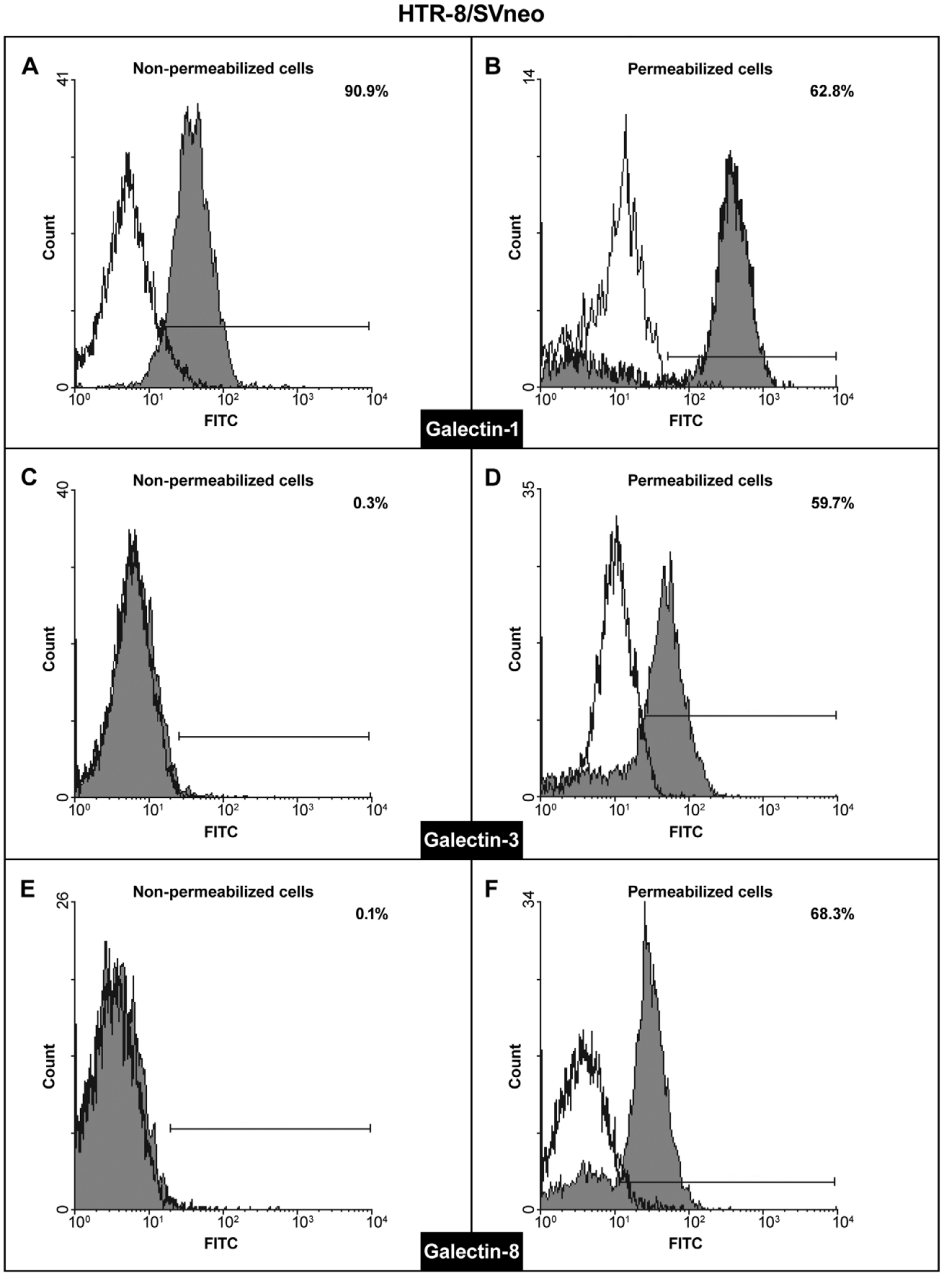
Cytofluorometrical analysis of the expressed galectin proteins in HTR-8/SVneo cells. The *x*-axis indicates fluorescence intensity measured on a log10 scale, and the *y*-axis indicates event counts on a linear scale. Specific staining is shown as gray, and non-specific staining (isotype-matched control IgG) as white histograms. Either non-permeabilized (A, C, E), or permeabilized cells (B, D, F) were probed for expression of gal-1 (A, B), gal-3 (C, D) and gal-8 (E, F) using appropriate antibodies. The percentages of non-permeabilized and permeabilized gal-1, -3 and -8 positive cells are shown in each histogram. A representative experiment of three is shown.

In order to comprehend fully the relevance of these results we wished to establish the galectin profile of the invasive cytotrophoblast. To that end RT-PCR was conducted using published primer sequences for human galectins 1–4, 7–10, 12–14 [45] on isolated cytotrophoblast (Fig. 6A) and the HTR-8/SVneo cell line (Fig. 6C). Both trophoblast cell types expressed RNAs for gal-1, gal-3 and gal-8, while gal-13 could be occasionally detected in isolated cytotrophoblast only. The finding of gal-13 was established (by subfractioning the trophoblast containing Percol layer) to result from occasional contamination of cell preparations with fragments of placental syncytiotrophoblast (data not shown). In order to evaluate relative abundances of transcripts for each of the three galectins found consistently in both cell types, real-time PCR was conducted and the RNA levels were normalized to GAPDH RNA (Fig. 6B, D, E). In isolated cytotrophoblast high levels of gal-3 and gal-8 RNAs were detected compared to gal-1 (Fig. 6B), while the opposite was the case for HTR-8/SVneo (Fig. 6D) where gal-1 RNA was the most abundantly expressed galectin RNA. Expression at the protein level by either cell type was examined and confirmed by Western blot of whole cell lysates (Fig. 6F). The bands present were of the expected molecular mass for each galectin with differences in band intensities between the cell types. In both cell types the molecular mass of gal-1 was 14 kDa, of gal-3 it was 30 kDa, while gal-8 was detectable at 36 kDa. Based on data from other systems, each of the expressed galectins has been proposed to have both intracellular and extracellular functions, which in some cases are shared between them. In order to determine whether localization of the galectins expressed was compatible with their participation in cell invasion, FACS analysis of gal-1, gal-3 and gal-8 was performed on permeabilized and non-permeabilizied cytotrophoblast (Fig. 7) and HTR-8/SVneo cells (Fig. 8). Isolated cytotrophoblast cells were released from placental tissue by sequential trypsin digestion, purified on a Percol gradient, and analyzed upon purification to deplete CD45 positive cells, also known to express galectins (as described in Material and Methods). Staining for galectins of unpermeabilized cells (A, C, E) was indicative of extracellular localization, while positivity in permeabilized cells (B, D, F) showed their presence in the cytoplasm. The results obtained show that all three galectins are present in the cytoplasm of isolated cytotrophoblast, as expected from the Western blot results. The relative distribution for each galectin differed, with gal-1 present in 74% of cells (Fig. 7B), gal-3 in 45% (Fig. 7D) and gal-8 in 34% (Fig. 7F). However, the data regarding the presence of these galectins at the cytotrophoblast cell membrane were somewhat surprising, since the only galectin found extracellularly was gal-1 in 32% of cells (Fig. 7A). Virtually no gal-3 was present, as only 0.1% of cells were stained (Fig. 7C), while 2.6% stained for gal-8 (Fig. 7E). Nevertheless, this result could explain the relatively large inhibition of cytotrophoblast invasion observed with anti-gal-1 in a cell type that expresses multiple galectins. The same analysis was performed on HTR-8/SVneo cells (Fig. 8). Intracellular localization was found for all three galectins, gal-1 in 63% cells (Fig. 8B), gal-3 in 60% (Fig. 8D) and gal-8 in 68% (Fig. 8F). Extracellularly, however, the only galectin found was gal-1 in 92% cells (Fig. 8A). Extracellular gal-3 and gal-8 were detected in 0.3% and 0.1% respectively (Fig. 8C, D).

## Discussion

To the best of our knowledge this is the first study to address the issue of the galectin profile of human invasive trophoblast cells and the functional relevance of any galectin for trophoblast invasion. The functional studies reported here have confirmed that gal-1 participates in trophoblast cell invasion. When endogenous gal-1 was blocked by antibody, cell invasion was significantly decreased, while additional recombinant gal-1 stimulated cell invasion. The data presented confirm expression of the three galectins, gal-1, gal-3 and gal-8, and provide cellular localization for each, which has not been reported so far for human invasive trophoblast cells.

Interactions of glycoconjugates with endogenous lectins such as galectins, have been long proposed to participate in several reproductive processes including fertilization, blastocyst attachment, and implantation [53]. Previous seminal studies that addressed the physiological significance of galectins for reproduction were conducted in mice with gene neutralization of gal-1 alone [25], or with gal-3 [27]. A long standing interest of this group has been the relevance of galectins for the invasive properties of normal and transformed trophoblast. Gal-1, gal-3, and gal-8 are expressed by the invasive extravillous trophoblast of the cell column [18], [19], [23] and the immortalized invasive trophoblast cell line HTR-8/SVneo [36]. Therefore, in the present investigation the issue of potential participation of gal-1 in human trophoblast cell invasion *in vitro* using cell models was examined. This approach was based on the availability of a unique anti-gal-1 antibody with function blocking characteristics demonstrated in other systems [47], as well as two molecular forms of biologically active recombinant human gal-1 [51]. In neurobiology, application of the same anti-gal-1 antibody with neutralizing activity strongly inhibited axonal regeneration of peripheral nerves after axotomy, both *in vivo* and *in vitro* [47]. In order to use this antibody to block cell function of the trophoblast, it was initially tested for recognition of gal-1 at the previously described placental locations. It was found to recognize gal-1 at all sites and cell types as previously described, and also to bind to HTR-8/SVneo cells in culture. Therefore, it was concluded that the anti-gal-1 used here met the initial criteria for further use to block cell functions.

Cell adhesion in general depends on the presence of membrane constituents able to bind stably to different surfaces. Since we have previously demonstrated that lactose, an inhibitory sugar for the lectin-type interaction of galectins, reduced invasion by the extravillous trophoblast HTR-8/SVneo cell line [36], its possible effect on cell adhesion was examined here. We hypothesized that endogenous gal-1 could participate in cell adhesion by binding cell membrane glycoproteins and/or ECM proteins produced by cells or present in the culture vessel coating. This was confirmed by our finding that lactose inhibited HTR-8/SVneo cell adhesion to plastic and collagen type I. Antibody that binds this gal-1 could in turn interfere with the efficiency of cell adhesion. In the presence of anti-gal-1 antibody, cell adhesion was increased to both plastic and ECM protein coated surfaces. This was an intriguing finding which probably reflects complex interactions between endogenous gal-1, its trophoblast ligands, and the antibody introduced into the test system. There is a possibility that the antibody reinforces existing complexes and adhesion, and/or further cross links membrane and ECM bound gal-1. A similar effect of the function blocking antibody could result from elimination of potential steric hinderance induced by gal-1, which, however, was not studied here. In an *in vitro* assay using melanoma cells, recombinant gal-1 increased attachment to laminin in a dose-dependent manner, which could be abolished by lactose [54] and anti-gal-1. In a smooth muscle cell model chemical cross-linking of gal-1 to β1 integrin indicated that dimeric gal-1 bound to a single molecule of β1 integrin and did not cross-link two β1 integrin molecules [11]. Nevertheless, this binding increased the availability of β1 integrin subunits on the cell surface and their activation. By analogy, endogenous cell surface gal-1 of HTR-8/SVneo cells could be bound to β1 integrin and potential further linking of gal-1 ligated membrane and ECM glycoproteins could be achieved by the antibody, possibly activating additional adhesive mechanisms. Gal-1 does not have specific receptors, but possible ligands or counter-receptors include ECM glycoproteins, as well as membrane glycoproteins, such as integrins. Cell column trophoblast cells *in vivo* [55], [56] and HTR-8/SVneo cells (our data, not shown) produce ECM proteins including laminin and oncofetal fibronectin. The invasive cytotrophoblast and HTR-8/SVneo cells also express the integrin receptors for these ECM proteins [55], [57], [58]. Both classes of glycoproteins are heavily glycosylated and identified as ligands for gal-1 in several systems and placenta [13]. Therefore, participation of gal-1 in cell adhesion and invasion is likely, possibly through formation of supramolecular structures as has been described for gal-1 on T cells, and other members of the galectin family, such as gal-3 [59].

It has been found that gal-1 modulates proliferation of different types of cells. The effects of gal-1 on cell proliferation are multifaceted and can be positive or negative, depending on the cell line and/or subcellular localization. The data presented here show increased absorbance in MTT test, suggesting stimulation of cell proliferation in HTR-8/SVneo cells, induced by exogenous recombinant gal-1. This, however, was not accompanied with change in abundance of proliferating or apoptotic cells. In some other responsive cell types gal-1 effects have been dissociated from proliferation and alterations in cell cycle. It has been shown in T cells that gal-1 can support cell survival without promotion of cell proliferation [60]. However, gal-1 isolated from placental tissue inhibited proliferation of choriocarcinoma cell line BeWo cells and reduced cellular uptake of BrdU at concentrations as high as 30 µg/ml and 60 µg/ml [61]. In BeWo cells the signaling cascade of gal-1 at high concentrations is proposed to include receptor tyrosine kinases JAK2, RET and VEGFR3 [62]. Apparently, the gal-1 growth promoting effect is cell-type specific, concentration sensitive, and could be both carbohydrate-dependent or -independent.

Availability of gal-1 is shown here for the first time to be important for trophoblast invasion *in vitro*. Since blocking endogenous gal-1, as well as supplementation with recombinant gal-1, is directly correlated with cell invasion, we propose that gal-1 functions as an important constituent of the molecular profile of invasion competent human trophoblast. The stabilized form of gal-1 (CS-gal-1) was more potent in inducing invasion, as a 1000 times higher concentration of Ox-gal-1 was required for a significant increase, indicating the relevance of lectin type interactions in this process. A molecular form dependent effect of gal-1 was described in an axonal regeneration model [51]. Our finding of gal-1 stimulation of human trophoblast could seem contradictory to the reported mouse knock-down data, but increasing data in the literature suggest that species-specific glycotypes exist [63], which could consequently be reflected by species specific relevance of their binding proteins, such as galectins. On the other hand, gal-1 is increasingly associated with invasiveness of cancer cells. Increased expression of gal-1 in hepatocellular carcinoma is significantly linked to the presence of metastasis [64]. We have previously shown that gal-1 is overexpressed in trophoblast of choriocarcinoma, invasive mole and placental site trophoblastic tumor [35]. Knocking down gal-1 with small interfering RNA in highly invasive cancer cells reduced invasion [38], [65]. Overexpression of gal-1 in an oral carcinoma cell model resulted in an increase of cell migration and invasion in low metastatic cancer cells *in vivo* and *in vitro* implicating matrix metalloproteinase (MMP-2 and MMP-9) expression [38], which however was not shown for HTR-8/SVneo cells (our unpublished observation). The finding that gal-1 is regulated by hypoxia in colorectal cells [66] is of potential significance for trophoblast. In early pregnancy physiological trophoblast invasion is achieved in hypoxic conditions, and sensitivity of trophoblast functions to HIF has been documented [67]. Gal-1-induced migration and invasion in hepatoma HuH-7 cells were mediated by the stimulation of Syk phosphorylation [66]. However, pathways and the effector molecules involved in gal-1 stimulation of normal human trophoblast invasiveness remain to be established. In general, exchange of receptors between membrane domains is a critical aspect of cellular sensitivity to extracellular cues. For EGFR and other cytokine receptors (including TGFβR, PDGFR, and IGFR), receptor recruitment to the galectin lattice potentiates ligand induced clustering and signaling [68]. There is a possibility that the same holds true for trophoblast and yet unidentified growth factor/cytokine receptors, so that altered/reduced accessibility of these receptors caused by anti-gal-1 antibody would consequently decrease signaling by serum or autocrine factors, for instance in cell invasion, as shown here. Conversely, increased availability of soluble gal-1 could result in its binding to additional membrane and/or ECM ligands and further crosslinking leading to increased invasion.

Since binding characteristics are shared among the members of the galectin family and their cellular functions may overlap, we undertook to determine the galectin localization in isolated trophoblast of first trimester of pregnancy and the established trophoblast cell line HTR-8/SVneo. Among the 11 galectins tested, galectins 1,-3, and -8 were consistently present in both trophoblast cell types, at mRNA and protein levels. However, their cellular distribution strikingly identified gal-1 as the only one with extracellular localization, which could possibly explain the considerable influence of antibodies to gal-1 on trophoblast invasion. The finding that the profile obtained is the same for cells of the established cell line detached without trypsin and trophoblast cells isolated from tissue using trypsin reduces the possibility that the absence of extracellular gal-3 and gal-8 was induced by the procedure used. Multiple galectins are also present in other tissues such as endometrium and decidua, endothelium, ovary cells, etc [20], [45], [69]. The same three galectins are expressed in human embryonic kidney HEK-293 and human foreskin fibrobasts [70]. The influence of galectin binding to membrane proteins and integrins in particular has been well studied and documented for gal-1, gal-3 and gal-8. In multiple cell lines gal-8 binding to different integrins inhibited cell adhesion [71], but the effect was not shared by gal-1 or gal-3 in the same cell types. Obviously, there is a need for further investigation of specific functions of each of the galectins expressed by the invasive trophoblast.

The dynamics and nature of binding partners for gal-1 responsible for the effect on trophoblast cell invasion remain to be elucidated in the future. A very complex picture is likely to emerge, given the expression of multiple galectins and gal-1 in particular by distinct, yet often adjacent cell types present within the placental bed.
